# Effects of Shear Stress on Production of FVIII and vWF in a Cell-Based Therapeutic for Hemophilia A

**DOI:** 10.3389/fbioe.2021.639070

**Published:** 2021-03-01

**Authors:** Brady Trevisan, Alshaimaa Morsi, Julio Aleman, Martin Rodriguez, Jordan Shields, Diane Meares, Andrew M. Farland, Christopher B. Doering, H. Trent Spencer, Anthony Atala, Aleks Skardal, Christopher D. Porada, Graça Almeida-Porada

**Affiliations:** ^1^Fetal Research and Therapy Program, Wake Forest Institute for Regenerative Medicine, Wake Forest School of Medicine, Winston-Salem, NC, United States; ^2^Faculty of Medicine, Zagazig University, Zagazig, Egypt; ^3^Aflac Cancer and Blood Disorders Center, Children’s Healthcare of Atlanta and Department of Pediatrics, Emory University, Atlanta, GA, United States; ^4^Department of Medicine, Section on Hematology and Oncology, Wake Forest School of Medicine, Winston-Salem, NC, United States

**Keywords:** FVIII, vWF, microfluidics, gene therapy, mRNA, shear stress, miRNA

## Abstract

Microfluidic technology enables recapitulation of organ-level physiology to answer pertinent questions regarding biological systems that otherwise would remain unanswered. We have previously reported on the development of a novel product consisting of human placental cells (PLC) engineered to overexpress a therapeutic factor VIII (FVIII) transgene, mcoET3 (PLC-mcoET3), to treat Hemophilia A (HA). Here, microfluidic devices were manufactured to model the physiological shear stress in liver sinusoids, where infused PLC-mcoET3 are thought to lodge after administration, to help us predict the therapeutic outcome of this novel biological strategy. In addition to the therapeutic transgene, PLC-mcoET3 also constitutively produce endogenous FVIII and von Willebrand factor (vWF), which plays a critical role in FVIII function, immunogenicity, stability, and clearance. While vWF is known to respond to flow by changing conformation, whether and how shear stress affects the production and secretion of vWF and FVIII has not been explored. We demonstrated that exposure of PLC-mcoET3 to physiological levels of shear stress present within the liver sinusoids significantly reduced mRNA levels and secreted FVIII and vWF when compared to static conditions. In contrast, mRNA for the vector-encoded mcoET3 was unaltered by flow. To determine the mechanism responsible for the observed decrease in FVIII and vWF mRNA, PCR arrays were performed to evaluate expression of genes involved in shear mechanosensing pathways. We found that flow conditions led to a significant increase in KLF2, which induces miRNAs that negatively regulate expression of FVIII and vWF, providing a mechanistic explanation for the reduced expression of these proteins in PLC under conditions of flow. In conclusion, microfluidic technology allowed us to unmask novel pathways by which endogenous FVIII and vWF are affected by shear stress, while demonstrating that expression of the therapeutic mcoET3 gene will be maintained in the gene-modified PLCs upon transplantation, irrespective of whether they engraft within sites that expose them to conditions of shear stress.

## Introduction

Hemophilia A (HA) is an X-linked genetic disorder caused by mutations in the factor VIII (FVIII) gene, resulting in the lack of functional clotting protein FVIII (Franchini and Mannucci, 2013). In plasma, FVIII circulates tightly bound to von Willebrand factor (vWF), a large multimeric glycoprotein produced in endothelial cells and megakaryocytes (McGrath et al., 2010).

The interaction between vWF and FVIII plays a critical role in the function, stability, and clearance of FVIII protein (Pipe et al., 2016), with changes in vWF concentration affecting overall FVIII levels (Lollar et al., 1988). For instance, the half-life of FVIII in the absence of vWF, as is the case in von Willebrand Disease, is around 3 h, while in the presence of vWF, FVIII half-life is 12 h (Over et al., 1978). The presence of vWF also plays a role in FVIII immunogenicity by shielding the epitopes of FVIII that common inhibitors recognize and preventing the uptake of FVIII by antigen-presenting cells (Dasgupta et al., 2007; Delignat et al., 2012).

We have previously reported that human placental cells (PLC) constitutively produce vWF (Morsi et al., 2018) and FVIII (El-Akabawy et al., 2020), and we developed a potential cell therapy platform by transducing human PLC with a lentiviral vector encoding a myeloid-codon-optimized (mco), bioengineered FVIII transgene that contains high-expression elements from porcine FVIII (ET3) (PLC-mcoET3) (El-Akabawy et al., 2020). Moreover, PLC-mcoET3 secreted FVIII protein with functional procoagulant activity at therapeutic and clinically meaningful levels (El-Akabawy et al., 2020). In addition, after prenatal administration, these cells are thought to lodge in perivascular sites, such as the liver sinusoids (Almeida-Porada et al., 2017), and therefore are subjected to shear stress from fluid flow. vWF has been shown to respond to fluidic shear stress by changing conformation, binding affinity, and protein activity (Vergauwe et al., 2014), but whether and how shear stress affects the amount of vWF secreted has not been explored. Moreover, the role of shear stress from fluid flow on FVIII production and secretion, to our knowledge, has not thus far been described.

These studies used a microfluidic platform designed to generate physiologically relevant levels of shear stress, to investigate how fluidic flow impacted the production and secretion of vWF, FVIII, and mcoET3 in PLC-mco-ET3. The results showed that shear stress did not affect levels of mcoET3, but downregulated the production of both FVIII and vWF in PLC-mco-ET3. In addition, the studies provided new mechanistic insights into how shear stress can decrease the production of both FVIII and vWF by increasing miR-30c and miR-10a *via* Kruppel-like Factor 2 (KLF2) (Wang et al., 2006), through mechanotransduction by integrins *via* the SRC and SHC1 proteins. These findings are of particular importance with respect to using the gene-modified PLCs for therapy, as they show that mcoET3 expression will persist unabated, irrespective of whether the transplanted PLCs engraft within regions that expose them to conditions of shear stress.

## Materials and Methods

### Culture and Transduction of Human Placental Mesenchymal Cells

Human placentas were obtained from full-term deliveries after informed consent, according to the guidelines from the Office of Human Research Protection at Wake Forest Health Sciences. Primary placental mesenchymal cells (PLC) were isolated from the placental chorionic layer after enzymatic digestion and grown in placental cell growth media (PCGM) consisting of α-minimum essential medium (α-MEM) supplemented with 17% AmnioMAX Basal Media, 15% fetal bovine serum (FBS), 2% AmnioMAX Supplement, 1% GlutaMAX, and 2.5 mg/mL gentamicin (ThermoFisher Scientific, Wilmington, DE, United States) (El-Akabawy et al., 2020). PLC were transduced with an HIV-based lentiviral vector encoding a myeloid codon-optimized (mco) B domain-deleted human/porcine hybrid FVIII transgene (ET3), termed mcoET3, at a multiplicity of infection of 10 transducing units/cell (El-Akabawy et al., 2020). Transduced cells (PLC-mcoET3) were passaged at 70–80% confluence using TrypLE (ThermoFisher Scientific, Wilmington, DE, United States) and PLC-mcoET3 were used in these studies after they had been passaged a minimum of three times post-transduction to ensure the absence of any episomal vector. Vector copy number (VCN) was determined using the Lenti-X proviral quantitation kit (Takara Bio, Mountain View, CA, United States) according to the manufacturer’s instructions, and established that PLC-mcoET3 had a VCN of 0.93.

### Manufacturing of Polydimethylsiloxane

### Microfluidics Devices

Single-use microfluidic devices were manufactured as previously described (Aleman et al., 2019) using polydimethylsiloxane (PDMS) (10:1 Sylgard 184 silicone elastomer and curing agent, respectively, Dow Corning, Midland, MI, United States). AutoCAD was used to design a 2-dimensional layout of channels that were 0.9 mm wide by 15 mm long. Two layers of double-sided tape (3 M, Maplewood, MN, United States) were then pressed onto a glass slide (VWR, Radnor, PA, United States) to reach the desired channel height of 200 μm. The tape was then laser cut into the designed layout and the outer tape was removed to leave a negative mold of the device channels. The glass slide was then taped around the perimeter to a sheet of aluminum foil that was folded up to create a boat that could hold the PDMS prior to polymerization. PDMS was then dispensed over the mold and allowed to cure to make the device with channels of the specified dimensions. Next, we covered a glass slide with the epoxy-based negative photoresist SU8 (MicroChem, Westborough, MA, United States) and pressed the PDMS device onto the SU8 to fill the channels. SU8 was solidified with UV light to make a permanent mold for production of more PDMS devices. A blunt needle was then used to punch inlet and outlet holes at the ends of the channels in the PDMS device, and the open side of the channels as well as a clean glass slide was then plasma-treated and permanently adhered to create a closed microfluidic device. The device was immediately seeded with cells by resuspending 300,000 PLC-mcoET3 in 200 uL of media, pipetting the cell suspension into a single channel, and incubating overnight to allow the cells to adhere to the channel and form a confluent monolayer to avoid subsequent proliferation. Alternatively, the device was first filled with 200 μL of 0.05% gelatin and allowed to solidify for 15 min, after which the excess gelatin was removed. The gelatin coating was used to provide a softer and more physiological environment for the cells. Tubing was then placed into the inlets and outlets and connected to a reservoir of media.

### Manufacturing of Polymethylmethacrylate Devices

Polymethylmethacrylate devices single-use microfluidic devices were manufactured by designing a 2-dimensional layout of the required channel, which was 50 mm long and 3 mm wide, and had a height of 1.5 mm corresponding to the thickness of the PMMA (McMaster-Carr, Elmhurst, IL, United States). This “channel layer” was fabricated by laser cutting the desired channel dimensions into a sheet of PMMA with a layer of double-sided tape adhered to either side. A solid sheet of PMMA (with no channels) served as the base layer, and the inlet layer was created by laser cutting holes in an additional sheet of PMMA to enable attachment of inlet and outlet tubing. These pieces were then sterilized with UV light (365 nm, 7 W cm^–2^) for at least 20 min. Subsequently, the base and channel layers were attached (with the double-sided tape) to form channels with an open top. The floor of this channel was coated with gelatin by pipetting 300 μL of 0.05% gelatin and allowing it to solidify for 15 min, after which excess gelatin was removed. This quantity of gelatin was necessary to prevent a meniscus effect that caused the center of the channel to remain bare of gelatin. The gelatin-coated device was seeded with 300,000 cells, which were allowed to attach overnight. This cell number was chosen to ensure the cells, upon attachment, formed a confluent monolayer to avoid subsequent proliferation. During this time, tubing was glued to the inlet and outlet holes of the inlet layer with epoxy. On the following day, when the cells had adhered to the floor of the channel, the inlet layer was attached to the top of the channel layer with double-sided tape to form a closed microfluidic device and the tubing connected to a separate well of 1.5 mL of media and hooked up to a peristaltic pump to flow media through the device to generate a set flow rate. Devices were discarded after use, and a new device was created for each experiment.

### Induction of Shear Stress on the Microfluidics Devices’ Cell Layers

Poiseuille’s law $\tau =\frac{6\,\eta \,Q}{h^{2}\,w}$ was used to determine the flow rate (Q) required to generate shear stresses (τ) of 0.5 or 0.05 dyne/cm^2^, given the height (h) and width (w) of the channel, and the viscosity (η) of 0.96 mPa⋅s to match previously measured cell culture media with similar levels of FBS. A four-channel precision micro peristaltic pump (Elemental Scientific, Omaha, NE, United States) was used to circulate cell culture media (1.5 mL) from the reservoir, through the device using 1.14 mm inner diameter tubing connected to the peristaltic pump, set at 10 and 100 rpm as determined to be required to generate the desired flow rate. The device was left in flow for 24 h before the cells and supernatant were collected for analysis.

### Computational Fluid Dynamics Analysis

The devices were created in Autodesk Fusion 360 (Autodesk, San Rafael, CA, United States) with a single solid piece to model each device, with a second separate piece created to model the media inside of the channel and in the inlet and outlet. The material of the device was set to acrylic and the material of the fluid was set to water to approximate the actual material of our devices and the properties of our media. A boundary condition of the flow rate applied by the peristaltic pump was set to the inlet and default settings were used to generate the mesh before the model was solved for the shear stress and visualized.

### Protein Quantification

Cell culture supernatant was collected at the 24-h time point, immediately centrifuged to remove cell debris, aliquoted, and frozen at −80°C. After supernatant collection, PLC-mcoET3 were harvested, counted as described below, and the cell number for each sample recorded. FVIII activity in the supernatant was quantified using aPTT assays, which were performed by the Wake Forest Baptist Medical Center Special Hematology Laboratory in accordance with standard clinical procedures, using a Top 300 CTS clinical coagulometer (Instrumentation Laboratories, Bedford, MA, United States). Quantification of vWF protein in the supernatants was determined by using a vWF-specific ELISA (ThermoFisher Scientific, Wilmington, DE, United States), as detailed in the Supplementary Material. The presence of interferon-γ in the supernatant was also measured using a high-sensitivity human ELISA Kit (assay range: 0.16−10.0 pg/mL) (ThermoFisher Life Technologies, Carlsbad, CA, United States), as detailed in the Supplementary Material.

### Cell Staining

Immediately after supernatant removal, cells were washed 3X with PBS (ThermoFisher Scientific, Wilmington, DE, United States) before fixing for 1 h in 4% paraformaldehyde (Electron Microscopy Sciences, Hatfield, PA, United States). To decrease background fluorescence, cells were treated with 0.1% sodium borohydride (Sigma-Aldrich, St. Louis, MO, United States) in PBS for 10 min, washed, and then blocked with blocking buffer [10% normal goat serum (NGS) in PBS], and finally washed with 2% NGS in PBS (working buffer). Mouse anti-human FVIII (Bio-Rad Laboratories, Hercules, CA, United States) at a dilution of 1:1,000 and rabbit anti-human vWF (Abcam, Cambridge, United Kingdom) at a dilution of 1:100 in working buffer were added, and the devices were placed on a shaker for 20 min before then being placed at 4°C overnight. The devices were washed with working buffer, and donkey anti-mouse Alexa Fluor 594 and mouse anti-rabbit Alexa Fluor 488 secondary antibodies (Invitrogen, Carlsbad, CA, United States) diluted 1:400 in working buffer were added to the devices and incubated for 30 min on a shaker. After washing with PBS, DAPI at a dilution of 1:1,000 was added for 5 min, followed by an additional wash before being coverslipped and imaged. The cells were then imaged in representative areas of each device using an Olympus BX30 microscope with a 10X objective.

### Image Analysis

DAPI images were imported into ImageJ and a threshold was run to remove the background fluorescence fully and leave only the cell nuclei. A particle analysis in ImageJ was performed to precisely quantify the number of cells that were present in the image. Three representative images for each sample were analyzed to achieve an average cell count within one frame, and this value was multiplied by a scaling factor to estimate the total number of cells present in each device. This total cell number was then used to normalize our protein production and secretion results to the number of cells present.

### Flow Cytometric Analysis of Cell Surface Integrins

Flow cytometric evaluation of PLC-mcoET3 was performed by staining 10^5^ cells per tube in 50 μL of BD Horizon Brilliant Stain Buffer with 10 μL of directly conjugated antibodies against CD61/Alexa Fluor 488, CD49a/PE, CD49e/BB700, and Integrin αvβ5/Alexa Fluor 647 (Becton Dickinson Biosciences, San Jose, CA, United States). Cells were washed with PBS with 0.1% Na Azide (Sigma-Aldrich, St. Louis, MO, United States) and centrifuged for 5 min at 1,500 rpm. The cells were resuspended in 500 μl of 1% paraformaldehyde (Electron Microscopy Sciences, Hatfield, PA, United States) in PBS. Background fluorescence was set using non-specific isotype-matched antibodies with respective fluorochromes. Cells were analyzed using a BD Accuri-C6 and data assessed using the FlowJo software using CD49a (Integrin α1) as selection criteria for PLC since 100% of the cells expressed this marker (Becton Dickinson Immunocytometry Systems, San Jose, CA, United States).

### RNA Extraction

RNA was extracted from devices immediately after the flow was stopped and the media removed, by adding RNAprotect (QIAGEN, Valencia, CA, United States) directly to the cells and scraping the gelatin layer with a pipette tip to ensure that all the cells were detached. The RNAprotect containing the cells was then collected and the RNeasy Mini Kit (QIAGEN, Valencia, CA, United States) was used as detailed in the Supplementary Material. Since sample contamination with gelatin present in the wells clogged the spin columns provided in the RNeasy kit, alternatively, samples were collected by adding TRIzol Reagent (ThermoFisher Scientific, Wilmington, DE, United States) directly to the cells, and RNA was isolated as detailed in the Supplementary Material. The use of TRIzol allowed high-quality RNA to be extracted at high yield (Alves et al., 2016). RNA integrity and purity were then verified using the Agilent RNA 6000 Nano Kit, as described in the Supplementary Material (Agilent Technologies, Santa Clara, CA, United States).

### Quantitative Reverse Transcription PCR

Quantitative reverse transcription PCR (RT-qPCR) was performed by generating cDNA from the collected RNA using the Omniscript RT kit (QIAGEN, Valencia, CA, United States), as described in the Supplementary Material. RNA was quantified using a NanoDrop 2000 (ThermoFisher Scientific, Inc., Wilmington, DE, United States), and RNA integrity was assessed using the Bioanalyzer RNA 6000 Nano assay and 2100 Bioanalyzer (Agilent, Santa Clara, CA, United States). RNA samples were then normalized to have a concentration of 20 ng/μl. 10 ng of DNA-free RNA was converted into cDNA using an Omniscript RT kit (Qiagen, Valencia, CA, United States). SYBR Green-based qPCR was conducted using PrimeTime qPCR Primer Assays (Integrated DNA Technologies, Inc., Coralville, IA, United States) using primers specific to: (1) the 3′ untranslated region (UTR) of FVIII (3′ UTR); (2) the modified FVIII transgene (mco-ET3); and (3) vWF. Human GAPDH served as an internal reference/housekeeping gene and was amplified using commercially available primers (Cat. Number PPH00150E, Qiagen, Valencia, CA, United States). The qPCR master mix was loaded into MicroAmp^TM^ Optical 96-well reaction plates and processed in the 7300 QuantStudio 3 RealTime-PCR system (Applied Biosystems, Foster City, CA, United States).

FAM-based qPCR using commercially available primers for KLF2, miR10a, miR30c, and GAPDH (ThermoFisher Scientific, Inc., Wilmington, DE, United States) was performed using TaqMan. TaqMan Fast Advanced master mix was loaded into MicroAmp^TM^ optical 96-well reaction plates and processed on a 7300 QuantStudio 3 RealTime-PCR system on the 96-well fast setting (Applied Biosystems, Foster City, CA, United States).

Fold-change of mRNA for a specific gene was calculated by the ΔΔCT method. First, the average CT of the housekeeping gene (GAPDH) for each sample was subtracted from the average CT of the gene of interest to obtain ΔCT. The average of the static ΔCT was calculated and subtracted from each individual static and flow ΔCT to obtain the individual ΔΔCT values. The fold-change was then calculated using 2^−ΔΔCT^. RT^2^ Profiler^TM^ Human Focal Adhesion PCR Array (QIAGEN, Valencia, CA, United States) was performed as described in the Supplementary Material to investigate the impact of shear stress on cell-extracellular matrix (ECM) adhesion.

### Statistical Analysis

Experimental results are presented as the mean plus/minus the standard error of the mean (SEM). All statistical analysis was performed using the R coding language in RStudio (RStudio, PBC, Boston, MA, United States). One-way ANOVA was employed for multiple comparisons. A *p* value <0.05 was considered statistically significant. Statistical analysis of the RT^2^ Profiler^TM^ PCR Array was performed using the RT^2^ Profiler RNA QC PCR Array Data Analysis Spreadsheet 1808 (Qiagen, Valencia, CA, United States).

## Results

### Manufacturing and Evaluation of Microfluidic Devices

**FIGURE 1:**
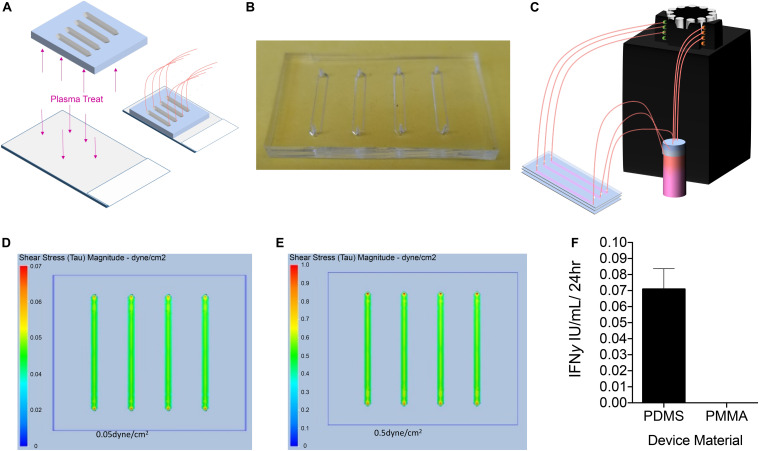
Design, Manufacturing, and Analysis of Microfluidic PDMS Devices. **(A)** To determine the effect of fluidic laminar shear stress on PLC transduced with a lentiviral vector encoding a myeloid codon-optimized bioengineered FVIII containing high expression elements from porcine FVIII (PLC-mcoET3), we designed and manufactured PDMS devices with the soft lithography method and adhered them to plasma-treated glass to form a closed channel. **(B)** Representative image of single-use PDMS device. **(C)** Upon completion, devices were connected to a reservoir of media and to a peristaltic pump to flow media through the devices to generate shear stresses of 0.05 or 0.5 dyne/cm^2^. **(D,E)** Computational fluid dynamics analysis showing shear stress maps at the base of the channels, demonstrating that PLC were experiencing either **(D)** 0.05 dyne/cm^2^ or **(E)** 0.5 dyne/cm^2^ throughout the channel, with a slight increase in the very center of the channel. **(F)** IFNγ secretion by PLC on microfluidic devices. PLC cultured on plasma-treated glass slides produced IFNγ while cells cultured on a PMMA device did not (*n* = 3).

To determine the effect of fluidic laminar shear stress on PLC transduced with a lentiviral vector encoding a mco bioengineered FVIII containing high expression elements from porcine FVIII (PLC-mcoET3), we manufactured several microfluidic devices designed to induce shear stresses similar to those generated in sites where PLC-mcoET3 lodge after prenatal transplantation (Almeida-Porada et al., 2017), such as the liver sinusoids. Specifically, we wanted to investigate whether shear stresses affected constitutive production and secretion of FVIII and vWF and/or expression of the mcoET3 transgene by PLC-mcoET3. The first microfluidics devices were fabricated using PDMS, as described in detail in the Materials and Methods and shown in Figures 1A,B. The devices were connected to a peristaltic pump (Figure 1C) to produce the necessary flow rates to reproduce the level of shear stress present within the liver sinusoids *in vivo*, assuming a flow velocity of 500 μm/s and a vessel inner diameter of 5 − 10 μm (Lalor and Adams, 1999). Poiseuille’s law was used to calculate the necessary flow rate in the devices to reach the desired level of shear stress, as described in the Materials and Methods. A static control device was manufactured to hold 1.5 mL of media, the same amount of media as the “in-flow” devices plus reservoir. After the media circulated within the device for 24 h, the supernatant was collected and the PLC-mcoET3 were stained and counted as described in the Material and Methods. Computational fluid dynamics was used to determine the levels of shear stress experienced by PLC. Analysis showed that PLC was experiencing either 0.05 or 0.5 dyne/cm^2^ throughout the channel, with a slight increase in the very center of the channel (Figures 1D,E).

Although these devices were effective in generating fluid flow at the desired rates, they often leaked from either the glass-PDMS interface or the tubing inlet and outlet. In addition, PDMS and glass have been reported to be able to initiate the activation of the intrinsic coagulation cascade, which could potentially lead to activation of FVIII in the supernatant of the cultured PLC-mcoET3, thereby skewing the measured FVIII activity (van Oeveren et al., 2002). Moreover, since placental tissues produce IFNγ upon stress (Banerjee et al., 2005) and IFNγ has been shown to have an effect on the production of tissue factor, which plays an important role in initiation of the coagulation cascade (van der Poll et al., 2001), we also used a high-sensitivity ELISA to test the culture supernatants of PLC-mcoET3 cultured in the devices to account for FVIII activation related to induced cell stress. Figure 1F shows that culturing PLC-mcoET3 on PDMS-on-glass led to an increase in IFNγ secretion (*n* = 3).

In order to overcome the limitations described above, we next coated the inside of the devices with gelatin. This resulted in uneven channel surfaces, irregular flow rates, and clogging. It also proved difficult to harvest cells or extract RNA from cells within devices, which was needed to analyze the effects of shear stress on the cells. These challenges led us to develop another device that would overcome the current issues.

**FIGURE 2:**
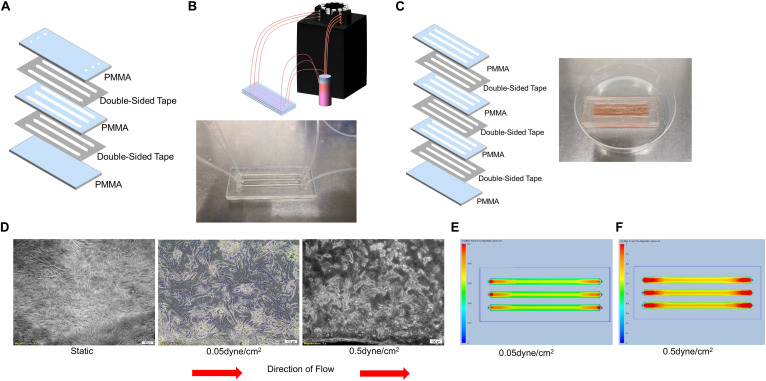
Design, Manufacturing, and Analysis of Microfluidic PMMA Devices. **(A)** PMMA devices were manufactured by adhering three laser-cut layers of PMMA and double-sided tape. **(B)** Representative image of single-use PMMA devices. These were connected to a reservoir of media and to a peristaltic pump to flow media through the devices to generate shear stresses of 0.05 or 0.5 dyne/cm^2^. **(C)** Static control devices were made by including multiple thicker channel layers to form a deep well to hold the same amount of media. Representative image of single-use static control PMMA devices. **(D)** Bright field images (5X magnification) of PLC in either static (left) or flow conditions (center and right). After PLC experienced shear stress of 0.05 dyne/cm^2^ for 24 h, they exhibited flow-induced changes in shape (center) with further changes in morphology seen after PLC experienced the higher shear stress of 0.5 dyne/cm^2^ for 24 h (right). **(E)** Computational fluid dynamics analysis showing shear stress maps at the base of the of channels, demonstrating that shear levels ranged from 0.05 dyne/cm^2^ at the edges to a slightly elevated shear stress of 0.06 dyne/cm^2^ in the center of the channel. **(F)** Computational fluid dynamics analysis showing shear stress maps at the base of the channels, demonstrating that the shear stress at the edges of the channel was 0.5 dyne/cm^2^, with a gradual increase to 0.7 dyne/cm^2^ in the center of the channel.

Polymethylmethacrylate devices has successfully been used in microfluidic devices to assess whole blood coagulation (Maji et al., 2018). Therefore, we next laser cut polymethyl methacrylate (PMMA) into several layers: a solid base layer, a layer with channels cut out, and a layer with holes at either side of the channel to use as inlets and outlets as is displayed in Figure 2A. These three layers of PMMA were held together using double-sided tape as described in the Materials and Methods. The need to easily collect cells, and RNA from the cells, within our devices led to the design of larger channels that would hold larger numbers of cells and could be opened to use like a well. The channels within these devices were 1.5 mm tall and 3 mm wide with a length of 50 mm and were coated with gelatin and seeded with 300,000 cells per channel (Figure 2B). The devices were hooked to the peristaltic pump (Figure 2B) to generate a flow rate able to produce the calculated shear stress of 0.05 and 0.5 dyne/cm^2^. Static control devices were also manufactured by adhering multiple 3 mm thick channel layers of PMMA on top of a single base layer to generate a well large enough to hold 1.5 mL of media (Figure 2C), the same amount of media used in the “in flow” devices. Evaluation of culture supernatants for the presence of IFNγ demonstrated that PLC-mcoET3 cultured in the PMMA devices did not secrete this cytokine (*n* = 3) (Figure 1F). Bright field images of the cells within the devices were taken after 24 h in either static or flow conditions. Static devices show a random distribution of PLC within the devices (Figure 2D, left). After PLC experienced shear stress of 0.05 dyne/cm^2^ for 24 h, they exhibited flow-induced changes in shape (Figure 2D, center). Further changes in morphology were seen in PLC that experienced the higher shear stress of 0.5 dyne/cm^2^ (Figure 2D, right).

Computational fluid dynamics analysis was used to determine the levels of shear stress that PLC was experiencing in the devices. Analysis of the PMMA devices under conditions that were used to generate about 0.05 dyne/cm^2^ was shown to create shear levels ranging from 0.05 dyne/cm^2^ at the edges to a slightly elevated shear stress of 0.06 dyne/cm^2^ in the center of the channel (Figure 2E). In PMMA devices under conditions set to generate 0.5 dyne/cm^2^, the variation was larger. Analysis showed that the shear stresses at the edges of the channel were 0.5 dyne/cm^2^, but this gradually increased to 0.7 dyne/cm^2^ in the center of the channel (Figure 2F). Of note is that all of the channels had increased shear stress in the area around the inlet and outlet of the devices, but this effect was not seen throughout the majority of the device.

### Evaluation of FVIII and vWF Secretion From PLC-mcoET3 Under Static and Shear Stress Conditions

**FIGURE 3:**
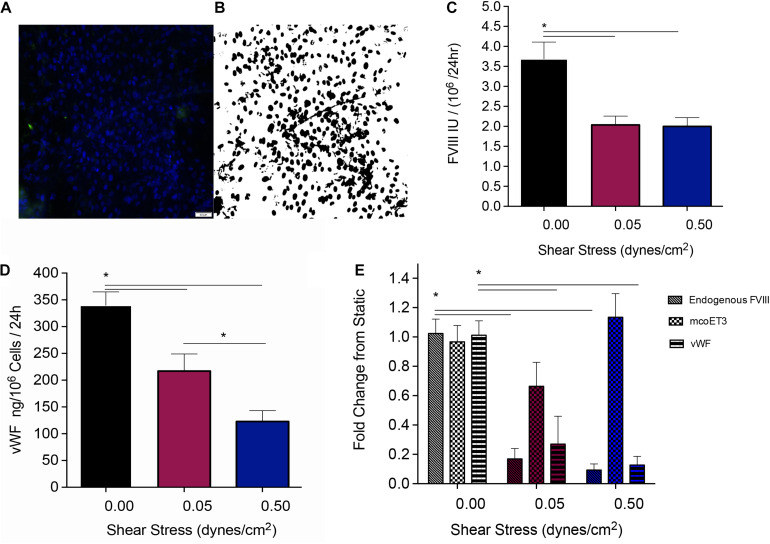
Quantification of the protein production and secretion from PLC-mcoET3 under conditions of shear stress. **(A)** Representative image of cell quantification: In order to quantify the number of cells in the devices, they were first stained with DAPI, and then **(B)** a black and white threshold was applied before a particle analysis was performed in ImageJ to count cells in representative areas. This count was scaled to the full area of the device channel and used to normalize our data to cell count. **(C)** fVIII/mcoET3 activity in PLC-mcoET3 supernatants was significantly reduced in conditions under flow when compared to static conditions (static conditions *n* = 15; 0.05 dyne/cm^2^ *n* = 11; and 0.5 dyne/cm^2^ *n* = 10) (*p* < 0.05). No difference in fVIII/mco-ET3 activity was found between shear stress conditions of 0.05 dyne/cm^2^ and 0.5 dyne/cm^2^. **(D)** ELISA was used to measure the vWF protein concentration in the supernatant (static conditions *n* = 15; 0.05 dyne/cm^2^ *n* = 9; 0.5 dyne/cm^2^ *n* = 8). vWF concentration in supernatants from PLC-mcoET3 under shear rates of 0.05 dyne/cm^2^ were significantly decreased when compared to those of under static conditions (*p* < 0.05). At the higher shear rate of 0.5 dyne/cm^2^, vWF concentration was significantly decreased when compared to both 0.05 dyne/cm^2^ (*p* < 0.05) and to static conditions (*p* < 0.05). **(E)** RNA expression of these proteins was examined with RT-qPCR performed in triplicate (endogenous FVIII *n* = 6; mcoET3 *n* = 5; and vWF *n* = 3). Endogenous/constitutive expression of both FVIII and vWF mRNA was decreased significantly at both shear rates (*p* < 0.05). However, mcoET3 mRNA expression was not affected by conditions of flow. *p < 0.05.

After PLC-mcoET3 was stably seeded in the channels of the devices, the media was changed and media was circulated through the devices at the shear stresses described above. After 24 h, the media was collected, briefly spun down, and frozen, to determine the FVIII activity as well as vWF content at a later time point. The inlet layer of the devices was then removed, and the cells stained with DAPI and imaged as shown in Figure 3A. These images were then analyzed in ImageJ (Figure 3B) as described in detail in the Materials and Methods to determine the total number of cells in each channel. The total number of cells in each channel was then used to normalize the quantification of proteins within the supernatant to 10^6^ cells. Quantification of fVIII/mco-ET3 activity in the supernatants was performed as previously described (El-Akabawy et al., 2020) using an activated prothrombin time (aPTT). PLC-mcoET3 under static conditions produced 3.69 ± 0.4 IU/10^6^ cells/24 h of fVIII/mco-ET3 (*n* = 15), but under the shear stresses of 0.05 and 0.5 dyne/cm^2^ fVIII/mco-ET3 secretion significantly reduced to 2.03 ± 0.2 IU/10^6^ cells/24 h (*n* = 11) and 1.99 ± 0.2 IU/10^6^ cells/24 h (*n* = 10), respectively (Figure 3C) (*p* < 0.05). However, no significant difference in fVIII/mco-ET3 was found in supernatants of cells subjected to 0.05 and 0.5 dyne/cm^2^ (Figure 3C). A human vWF-specific ELISA was used to determine vWF concentration in the supernatants of PLC-mcoET3 under static and fluidic flow conditions. In static settings, PLC-mcoET3 produced 339.9 ± 25 ng/10^6^ cells/24 h (*n* = 15) of vWF, but this concentration decreased significantly (*p* < 0.05) to 216.89 ± 32 ng/10^6^ cells/24 h in conditions of low shear stress (*n* = 9), and at a higher shear stress, vWF in supernatants was further reduced to 123 ± 20 ng/10^6^ cells/24 h (*n* = 9) (*p* < 0.05) (Figure 3D).

### Evaluation of FVIII, mcoET3, and vWF mRNA Under Static and Shear Stress Conditions

Quantitative reverse transcription PCR with primers specific to vWF, the FVIII 3′ UTR (to measure constitutive levels of endogenous FVIII mRNA), and to the mco-ET3 transgene was performed to determine whether the decrease in FVIII and vWF protein by conditions of flow was due to a decrease in mRNA. Figure 2E depicts fold-change in levels of vWF, FVIII, and mcoET3 mRNA when compared to static conditions. Endogenous FVIII mRNA decreased by 10-fold and 11.3-fold when cells were placed at a shear stress of 0.05 dyne/cm^2^ and 0.5 dyne/cm^2^, respectively, when compared to static conditions. In similarity, vWF mRNA decreased fivefold at the lower shear stress and 8.4-fold when cells were subjected to the higher shear stress. However, mcoET3 mRNA was not significantly affected by any conditions of flow (Figure 3E).

### Determining the Impact of Fluidic Shear Stress on PLC-mcoET3 Integrin Expression

**FIGURE 4:**
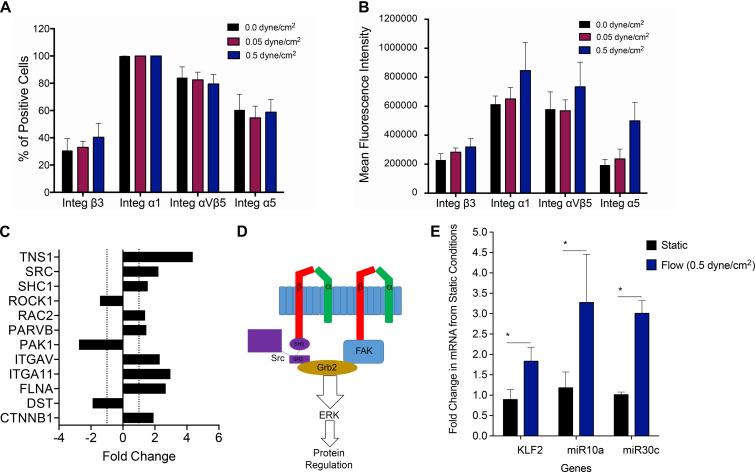
Investigating molecular pathways involved in mechanotransduction in PLC-mcoET3. **(A)** Flow cytometric analysis of integrins which have previously been reported to be affected by shear stress (*n* = 4 for each integrin). Shear stress did not affect the percentage of cells positive for any of the integrins studied. **(B)** Mean fluorescent intensity (MFI) obtained by dividing each integrin MFI by the respective isotype’s MFI (*n* = 4) was also used to investigate differences in cell surface integrin expression. No statistically significant differences were found between cells in static conditions and the cells under fluidic shear stress. **(C)** PCR array-based pathway-focused gene expression analysis on RNA isolated from the PLC-mcoET3 cultured under static conditions (*n* = 3) and conditions of shear stress (0.5 dyne/cm^2^) to examine possible shear-responsive pathways affected by shear stress (*n* = 3) demonstrating upregulation (+fold change) or downregulation (- fold change) of several genes related to shear-sensing pathways. **(D)** Mechanotransduction of the SRC pathway and FAK through beta integrins initiates the ERK pathway as a possible mechanism for the flow regulation of PLC-mcoET3. **(E)** RT-qPCR to quantify KLF2 (*n* = 6), miR10a (*n* = 4), and miR30c (*n* = 3) under static and flow conditions demonstrated that KFL2, miR10a, and miR30c are significantly increased under flow (*p* < 0.05). *corresponds to *p* < 0.05.

Cells sense mechanical stimuli, such as fluidic shear stress, through several mechanosensitive molecules including integrins (Martino et al., 2018). We, therefore, next investigated if and how conditions of shear stress impacted integrins expressed by PLC-mcoET3. We used flow cytometry to characterize the expression of integrins α1 (*n* = 4), α5 (*n* = 4), β3 (*n* = 4), and αvβ5 (*n* = 4), which have previously been reported to be affected by shear stress. Shear stress at 0.05 dyne/cm^2^ and 0.5 dyne/cm^2^ did not affect the percentage of cells positive for integrins α1, α5, β3, or αvβ5 (Figure 4A). To investigate if the levels of integrin expression were altered under flow, mean fluorescent intensity (MFI) was also calculated (Figure 4B), and no statistically significant difference in cell surface integrin expression was found between cells in static conditions and the cells under fluidic shear stress.

### Investigating Molecular Pathways Involved in Mechanotransduction in PLC-mcoET3

Although differences in integrin protein expression were not found to be statistically significant between static and the different fluidic shear conditions, the data showed that shear stress-sensitive integrins are present on PLC-mcoET3, suggesting that signaling through the expressed integrins could be responsible, at least in part, for the alterations observed in PLC-mcoET3 in conditions of flow. Therefore, we next performed PCR array-based pathway-focused gene expression analysis on RNA isolated from the PLC-mcoET3 cultured under static conditions and conditions of shear stress (0.5 dyne/cm^2^) to elucidate the molecular pathways responsible for the observed flow-induced alterations in expression of FVIII and vWF. Because integrity of RNA molecules is crucial to be able to replicate the gene expression signature at the precise moment of RNA extraction (Schroeder et al., 2006), the quality of all isolated RNA samples was tested on an Agilent 2100 Bioanalyzer, and only samples with an RNA integrity number (RIN) higher than eight were used.

The full results of this array analysis can be found in Supplementary Figure 1. Of interest was that, in addition to the upregulation/downregulation of several beta integrins, analysis of our focused array data also showed a 2.2-fold and a 1.5-fold increase in SRC and SHC1 gene expression, respectively (Figure 4C). These proteins are known to be involved in the process of shear mechanotransduction through their association with specific integrins in a pathway that uses shear mechanotransduction to initiate the ERK pathway through GRB2, as portrayed in Figure 4D. Importantly, this SRC/SHC1-driven activation of the ERK pathway has been shown to mediate the shear-dependent increase in levels of the zinc finger transcription factor lung Krüppel-like factor (KLF2) (Wang et al., 2006), which can regulate the transcription of vWF (Dekker et al., 2006). We therefore next performed RT-qPCR-based studies to investigate whether KLF2 was playing a role in the downregulation of endogenous expression of FVIII and vWF in PLCs and identify the responsible downstream effectors. As can be seen in Figure 4E, culture of PLCs in conditions of flow/shear stress (0.5 dyne/cm^2^) led to a statistically significant (*p* < 0.05) ∼2-fold increase in KLF2 mRNA levels compared to PLCs cultured in static conditions.

Shear stress has also been shown to induce the expression, nuclear accumulation, and association of retinoid acid receptor-α (RARα) and retinoid X receptor-α (RXRα). RARα and RXRα then serve as a “director” and an “enhancer,” respectively, to augment RARα binding to RA-responsive element (RARE) and thereby enhance miR-10a expression, in a KLF2-dependent fashion. Since GATA6 contains a target site for miR-10a (Lee et al., 2017) and GATA6 is known to bind to the promoter of vWF to activate its transcription (Wang et al., 2004), we next performed RT-qPCR to quantitate levels of miR-10a in PLCs cultured in static vs. flow conditions. These analyses Figure 4E showed a marked 3.3-fold upregulation of miR-10a (*p* < 0.05).

Next, we performed studies to investigate why exposure of PLC-mcoET3 to conditions of flow/shear stress markedly reduced the levels of mRNA for endogenous FVIII while having no effect on expression of the lentiviral vector-driven expression of mcoET3. Human FVIII and mcoET3 share greater than 90% identity at the nucleotide level, thus we reasoned that the differential response of these two FVIII molecules by flow must arise as a result of differences in their regulatory elements. A key difference between the endogenous/native FVIII transcript and that produced from the lentiviral vector is the absence of the 3′ untranslated region (UTR) in the vector-encoded mcoET3, which is derived from cDNA. Since in addition to miR10a, KLF2 upregulates various micro RNAs such as miR30c, and overexpression of miR-30c has been shown to decrease FVIII expression, we next examined the levels of miR30c in PLC-mcoET3 in static vs. flow conditions. These studies demonstrated that exposure to flow resulted in a nearly threefold induction of miR-30c message in PLC-mcoET3 (*p* < 0.05) (Figure 4E), providing a mechanistic explanation for the differential response of the endogenous/native FVIII and the mcoET3 transgene to conditions of flow.

## Discussion

Amid the rapid recent progress in HA therapies (DiMichele, 2018), FVIII transgene-modified cell platforms have arisen as a new class of promising biologicals (Shi et al., 2006; Porada et al., 2010; Doering et al., 2018). To fulfill their therapeutic promise, however, several challenging criteria must be met, including an optimized transgene that enables production and secretion of a functional FVIII molecule at therapeutic levels without inducing cellular stress responses (Zolotukhin et al., 2016). In addition, the gene-modified cells must possess the ability to efficiently produce and secrete FVIII, they should lodge/engraft and persist long-term within a broad range of tissues upon infusion, and they need to evade the recipient’s immune system, despite expressing a therapeutic protein that is perceived as foreign. We recently reported the development of a platform, based upon human PLC engineered with a lentiviral vector to express a mco human/porcine hybrid FVIII (ET3) molecule, PLC-mcoET3, that fulfills all of these criteria (El-Akabawy et al., 2020).

To restore hemostasis upon transplantation, the PLC-mcoET3 must lodge in perivascular sites to provide the secreted mcoET3 ready access to the circulation. As such, these cells will be placed in conditions of flow-induced shear stress, the biological effects of which have never before been explored in the context of FVIII transgene-modified cell platforms. PLC endogenously express low levels of FVIII. Moreover, they also express low levels of the FVIII carrier protein vWF, the conformation of which is known to be impacted by shear stress. Specifically, hydrodynamic forces have been shown to be responsible for extensive conformational transitions in the vWF multimers that change their globular form, a stretched structure, to a linear conformation, with flow acceleration favoring extension and thickening of vWF strands (Zheng et al., 2015). However, to our knowledge, the effect of flow on the expression of FVIII and vWF has not yet been explored. PLC-mcoET3 thus provide a unique paradigm in which to define the effects of flow-induced shear stress on the expression of both the transgene-encoded mcoET3 and the native FVIII locus, as well as the FVIII carrier vWF, and to delineate the molecular mechanism(s) driving any observed effects. For such studies to be possible, however, it is first necessary to culture PLC-mcoET3 in conditions of flow-induced shear stress that recapitulate those present within the anatomic sites of engraftment within the body.

As the liver sinusoids are the site where PLC-mcoET3 are thought to lodge after administration (Almeida-Porada et al., 2017), in the present studies, we designed, manufactured, and validated a cell culture device based on microfluidic technology to simulate the unique physiology of these structures. Specifically, we designed this device to accurately recapitulate the flow-induced shear stress to which resident cells are exposed in these minimal functional units of the liver. We then used this new device to obtain some of the first data to-date on the effects that these relatively low levels of shear stress exert on the levels of expression of FVIII and vWF in a novel cell therapy product (PLC-mcoET3) we have developed to treat HA. Previous studies have examined the effect of flow-induced shear stress on endothelial cells, however, it is important to note, that this prior work was designed to model the high shear present in the microcirculation (arterioles and capillaries), where the values of forces are typically >20 − 30 dyne/cm^2^. At these high shear stresses, vWF undergoes micro- and macro-conformational changes from a globular state to a stretched chain conformation, where especially the A1 domain undergoes structural changes relevant to its functionality (Schneider et al., 2007). Another study using similarly high shear stresses provided evidence that the expression of vWF may also be affected (Hough et al., 2008). The conditions within the liver sinusoids are markedly different, as flow rates and intravascular pressures within the venous circulation are much lower, and the resultant shear stress is on the order of 0.05−0.5 dyne/cm^2^. We, therefore, designed our microfluidic device to model these low shear stresses to enable us to gain insight into how the production and secretion of FVIII and vWF are affected by the physiological conditions that the cells synthesizing these coagulation proteins experience *in vivo*.

The molecular (RT-qPCR) and functional (aPTT) assays we performed herein demonstrated that the production and secretion of endogenous FVIII and vWF are both decreased under levels of shear stress present in the liver sinusoids, but the production of FVIII from our engineered mcoET3 transgene was unaffected by flow. The levels of vWF in the supernatant were further decreased when the shear stress rate was increased from 0.05 to 0.5 dyne/cm^2^ (still within the physiological range for the liver sinusoids), but the levels of FVIII were not further reduced beyond those seen at 0.05 dyne/cm^2^.

To elucidate the mechanism for this flow-induced downregulation of FVIII and vWF, we first used a pathway-focused PCR array to determine what shear responsive pathways are being activated in PLC-mcoET3. This analysis showed that both the SHC and SRC genes, which have both previously been shown to be a part of the β integrin-dependent cellular shear stress response (Arias-Salgado et al., 2003; Liu et al., 2008), are upregulated in PLC-mcoET3 exposed to flow conditions. Following its activation by flow-sensing β integrins, c-Src then binds to the SH2 domain of the Shc1 protein, triggering tyrosine phosphorylation of Shc1, and allowing it to bind growth factor receptor bound protein 2 (Grb2). This then leads to the activation of the extracellular signal-regulated kinase 2 (ERK2), Ras/mitogen activated protein (MAP) kinase, and ERK5 pathways (Jalali et al., 1998). Importantly, this Src/Shc1-driven activation of the ERK pathway has been shown to mediate the shear-dependent increase in levels of the zinc finger transcription factor KLF2 (Kumar et al., 2018) which in recent years, has emerged as a prime and pivotal candidate for directly relaying biomechanical shear forces into a gene transcription profile that might determine endothelial phenotype in response to flow (Dekker et al., 2002; van Thienen et al., 2006; Wang et al., 2006). This shear stress-induced upregulation of KLF2 expression is abrogated by blocking various β integrins or disrupting F-actin, supporting the notion that signaling/crosstalk between β integrins (expressed on human PLCs) and the actin cytoskeleton regulate KLF2 expression (Chu et al., 2019). Of particular note, other studies have provided evidence that KLF2 can regulate the transcription of several of the best-known endothelial signature genes, including vWF (Dekker et al., 2006). In agreement with these prior reports in endothelial cells, the present studies demonstrated that culture of PLC-mcoET3 in conditions of flow/shear stress present in liver sinusoids (0.5 dyne/cm^2^) led to a ∼2-fold increase in KLF2 mRNA levels compared to PLC-mcoET3 cultured in static conditions, supporting the conclusion that KLF2 was playing a role in the observed downregulation of endogenous expression of FVIII and vWF in PLCs. Moreover, these enhanced levels of KLF2 led to upregulation in expression of miR-10a, which is known to target GATA6, precluding it from binding to the vWF promoter to activate its transcription, ultimately leading to downregulation of vWF (Wang et al., 2004; Lee et al., 2017).

Other heavily up- or down-regulated genes could also be important to note. For example, in shear conditions, we found that TNS1 the gene encoding tensin 1, a key protein in cell adhesion, is markedly upregulated. Tensin 1 has been shown to be essential in the creation of ECM and has been shown to positively regulate cell migration (Bernau et al., 2017). As such, tensin could play a role in repair in damaged areas where shear stress is increased. The most down-regulated gene seen in our analysis was ITGA6, an alpha integrin. High expression of this integrin has been shown to enhance tumor cell invasion (Brooks et al., 2016), while its downregulation with miR-143-3p has been shown to suppress tumor growth (Jin et al., 2018). This could be further studied to determine if this miRNA is upregulated in shear stress and if this pathway could be harnessed to create a cell therapy or other treatment to prevent tumor growth. Another gene that was heavily downregulated is PAK1, which has previously been shown to be downregulated in shear stress by KLF2 through the ERK5 pathway, to inhibit endothelial migration (Komaravolu et al., 2015). Further studies of the focal adhesion genes that are influenced by shear stress can be performed to understand the mechanism and possible ways to harness these genes and interactions. Cells engrafted within the body, such as in the liver sinusoids, exist in a complex environment and are affected by many mechanical signals in addition to shear stress. These cues and signals, such as tissue stiffness, which could differ in physiological conditions compared to our devices, or cell orientation, which develops in physiological conditions, could alter the way that the cells experience shear stress or behave in response to it. The relevance of other mechanical signals and properties should be further studied to gain a complete understanding of the way that these forces, alongside shear stress, can regulate gene expression.

Having delineated a plausible pathway by which flow was decreasing vWF expression in the PLC-mcoET3, we next undertook studies to explain the seemingly paradoxical observation that exposure of PLC-mcoET3 to conditions of flow/shear stress markedly reduced the levels of mRNA for endogenous FVIII while having no effect on expression of the lentiviral vector-driven expression of mcoET3. Since mcoET3 shares greater than 90% identity with human FVIII at the nucleotide level, we reasoned that the differential response of these two FVIII molecules by flow must arise as a result of differences in their regulatory elements. A key difference between the endogenous/native FVIII transcript and that produced from the lentiviral vector is the absence of the 3′ untranslated region (UTR) in the vector-encoded mcoET3, which is derived from cDNA. A recent report of HA in human patients who lack F8 mutations, showed that overexpression of miR-30c decreased FVIII expression, while a miR-30c inhibitor partially restored FVIII expression in two cell lines that constitutively express FVIII (Jankowska et al., 2020a). These findings led the authors to hypothesize that expression of miRNAs targeting the 3’-UTR of FVIII mRNA can modulate FVIII levels and may be responsible for a FVIII-deficiency phenotype that clinically manifests as HA (Jankowska et al., 2020b). Because KLF2 overexpression has been shown to induce upregulation of the miR-30 family members miR 30b and 30c (Doebele et al., 2018), we next examined the levels of miR30c in PLC-mcoET3 in static vs. flow conditions and demonstrated that exposure to flow led to a nearly threefold induction of miR-30c in PLC-mcoET3, providing a mechanistic explanation for the differential response of the endogenous/native FVIII and the mcoET3 transgene to conditions of flow. These findings are of particular note with respect to using the gene-modified PLCs for therapy, as they suggest that mcoET3 expression will persist, even following engraftment of the transplanted PLC-mcoET3 within regions that expose them to conditions of shear stress. Collectively, the results of our focused array and RT-qPCR studies provide mechanistic insight into how shear stress can decrease the production of both FVIII and vWF through increasing miR-30c and miR-10a *via* KLF2 through mechanotransduction by integrins through the c-Src and Shc1 proteins.

In conclusion, we have developed a novel physiologically relevant microfluidic-based device that models the shear stress present within the liver sinusoids and have used this device to show, for the first time, that the low levels of shear stress present in this unique vascular bed down-regulate the production of both FVIII and vWF through a signaling cascade involving beta-integrins, c-Src, KLF2, and miRs. These newly defined shear stress-induced pathways could facilitate the identification of new targets that can regulate the production of both FVIII and vWF and thereby provide novel ways of treating HA and von Willebrand disease. Given the importance of underlying mechanical forces in the normal physiological response to maintain hemostasis (Spronk et al., 2003), there is no doubt that, in the coming years, the increasing use of microfluidics will allow for new and important findings in the fields of endothelial cell behavior, mechanotransduction pathways, coagulation protein production and behavior, and the testing of novel therapeutics *in vitro* (Trevisan et al., 2020).

## Data Availability Statement

The original contributions presented in the study are included in the article/Supplementary Material, further inquiries can be directed to the corresponding author/s.

## Author Contributions

BT, AM, JA, and MR performed experiments, data analysis, and interpretation. JA, DM, AF, and AS provided expertise. CD, HS, and AA provided reagents. BT drafted the manuscript. GA-P and CP approved final version of the manuscript. BT, GA-P, and CP conception and experimental design. GA-P and CP supervised experiments and secured funding. All authors contributed to the article and approved the submitted version.

## Conflict of Interest

The authors declare that the research was conducted in the absence of any commercial or financial relationships that could be construed as a potential conflict of interest.
