# Supplementary material for: Effects of Shear Stress on Production of FVIII and vWF in a Cell-Based Therapeutic for Hemophilia A
Source: Front Bioeng Biotechnol. 2021 Mar 1;9:639070. doi: 10.3389/fbioe.2021.639070 (PMC7957060; doi:10.3389/fbioe.2021.639070)
Supplement: Supplementary file 1 [file Table_1.DOCX]

Supplementary Material

**Figure Legend**

**Supplementary Figure 1.** PCR array-based pathway-focused gene expression analysis on RNA isolated from the PLC-mcoET3 cultured under static conditions (n=3) and conditions of shear stress (0.5 dyne/cm^2^) (n=3) demonstrating upregulation/downregulation of several genes related to shear-sensing pathways.

**Materials and Methods**

**Vector copy number (VCN) determination.** To determine the precise proviral copy number per diploid human genome, vector copy number was measured using the Lenti-X Provirus Quantitation Kit (Takara Bio USA, Inc. Mountain View, CA). First culture media was removed from the flask, cells were washed with PBS, and TrypLE (ThermoFisher Scientific, Wilmington, DE, USA) was added to the flask in order to detach the cells. An equivalent amount of tissue culture media was then added to the TrypLE cell-containing solution and cells were pelleted by 5 minutes centrifugation f at 1500 rpm. DNA was extracted from the cell pellet using a NucleoSpin Tissue kit by aspirating the liquid from the cell pellet and resuspending the pellet in 200μL of T1 Buffer. Then 25μL of Proteinase K solution and 200μL of Buffer B3 were added and the sample was incubated at 70˚C for 10 minutes. The DNA binding conditions were adjusted by adding 210μL of 100% ethanol, and the samples were vortexed to mix. The mixture was added to the NucleoSpin Tissue Column and the DNA was bound to the column by centrifuging for 1 minute at 11,000 x g and the flow-through was discarded. The column was then washed with 500μL of Buffer BW and centrifuged for 1 minute at 11,000 x g and washed a second time with 600μL of Buffer B5 and centrifuged for 1 minute at 11,000 x g with the flow-through discarded after each wash. The silica membrane was dried by centrifuging for 1 minute at 11,000 x g, and the DNA was eluted by adding 100μL of Buffer BE to the spin column for 1 minute before centrifugation at 11,000 x g. The eluted DNA was then stored at -20˚C until it was used. A master mix of the PCR reagents for the Takara Provirus Quantitation Kit was made by adding 6.8μL of PCR-grade water, 0.4μL of the Lenti-X Provirus Forward Primer, 0.4μL of the Lenti-X Provirus Reverse Primer, the ROX Reference Dye LMP, and 10μL of TB Green Advantage qPCR Premix. A standard curve was created by adding 2μL of the control template to 18μL of EASY Dilution buffer performing 1:5 serial dilutions 5 times. Two wells of only EASY Dilution buffer were used as no template controls. The DNA samples were then diluted in EASY Dilution buffer to 50ng/μL in a 20μL sample, and then each sample was serial diluted 1:5 two times. The PCR plate was then made by adding 18μL of master mix, and 2μL of each sample. The standards were run in duplicate and the samples were run in triplicate. PCR parameters included an initial denaturation at 95˚ for 30 seconds, 40 qPCR cycles consisting of 5 seconds at 95˚C and 31 seconds at 60˚C, followed by a dissociation curve with 15 seconds at 95˚C followed by 1 minute at 60˚C and 15 seconds at 95˚C. The data was then analyzed by generating a standard curve with the C_T_ values plotted against the concentration of the standard dilutions. The qPCR copy number equivalent was determined by creating a trend line along the standard curve and qPCR copy number was determined by plugging the C_T_ values for each sample in the trend-line equation. The qPCR copy number equivalent was then normalized for each dilution by the dilution factor, and was converted to a provirus copy number by multiplying the qPCR copy number by 62.84 provirus copies/qPCR copy. The cell number was determined using the generic conversion of 6.6pg of gDNA/cell to approximate 15,151.51 cells per sample, and the provirus copy number was divided by this cell number to determine provirus copies per cell.

**vWF ELISA.** Quantification of vWF protein in the supernatants was determined by using a vWF-specific ELISA (ThermoFisher Scientific, Wilmington, DE, USA). First, the lyophilized standard resuspended in 440μL of 1x Assay Diluent B and gently mixed. The standard solution was serially diluted 6 times by adding 200μL of the standard to 300μL of 1x Assay Diluent B. 100μL of each standard and 100 μL of each of the undiluted supernatants were added to individual wells of the ELISA plate and incubated at room temperature for 2.5 hours. The solution was discarded and the plate was washed 4 times with 300μL 1x washing buffer. 100μL of the biotin conjugate was added to each well and the plate was incubated at room temperature on a shaker for 1 hour. The biotin solution was discarded and the plate was washed 4 times. Streptavidin-HRP solution was added (100μL) to each well and incubated for 45 minutes on a shaker at room temperature. The Streptavidin-HRP solution was then discarded and the plate was washed 4 times. Then 100μL of TMP Substrate was added to each well and the plate was incubated for 30 minutes, on a shaker, in the dark, at room temperature. Finally, 50μL of Stop Solution was added and the side of the plate was gently tapped to mix. The absorbance was then immediately read at 450nm. The standard was used to create a standard curve and the equation of the trend-line was used to determine the vWF concentration from the optical density.

**Interferon Gamma ELISA**. The presence of interferon-γ in the supernatant was measured by sing a high-sensitivity human ELISA Kit ThermoFisher Life Technologies, Carlsbad, CA To perform he Interferon Gamma ELISA the standard was reconstituted in ultrapure water and serial diluted 5 times by adding 160μL of standard to 240μL of ultrapure water. Regarding the ELISA plate, first 50μL of Biotinylated Antibody Reagent was added to each well. Then 50μL of each standard or sample was added to each well and the plate was covered with an adhesive plate cover before incubating for 2 hours at room temperature. The plate cover was then removed and washed 3 times with wash buffer. Then 100μL of the prepared Streptavidin-HRP solution was added to each well and a new adhesive plate cover was used to seal the plate before incubating for 30 minutes at room temperature. The plate cover was then removed and the plate was washed 3 times. 100μL of TMB substrate was added to each well and the plate was developed in the dark for 30 minutes at room temperature before 100μL of stop solution was added to each well. The absorbance was then immediately read at 450nm and 550nm, and the 550nm values were subtracted from the 450nm values. A standard curve was generated, and a trend-line associated with the standard curve was used to calculate interferon gamma concentration from the optical density.

**RNA Isolation.** RNA was isolated using the TRIzol Reagent (ThermoFisher Scientific, Wilmington, DE, USA) by adding 300μL TRIzol directly to the cells in the microfluidic device and scraping the bottom of the device with a pipette tip. The cell-containing solution was removed from the device and placed in a 15mL tube where 200μL of TRIzol was added and mixed with pipetting before it was allowed to incubate for 5 minutes. After 5 minutes, 250μL of chloroform was added and the sample was incubated for 3 minutes. The sample was then centrifuged for 15 minutes at 12,000 x g at 4˚C. The aqueous phase containing the RNA was then removed to a new 15mL tube and 10μg of glycogen was added to the solution. 250μL of isopropanol was added to the aqueous phase and incubated for 10 minutes, before centrifuging for 10 minutes at 12,000 x g at 4˚C. The supernatant was gently discarded with a micropipettor and the pellet was resuspended in 1mL of 75% ethanol and vortexed. The sample was then centrifuged for 5 minutes at 7500 x g at 4˚C, and the supernatant was gently discarded with a micropipettor. The pellet was allowed to air dry for 10 minutes, ensuring the pellet did not fully dry, and was resuspended in 30μL of RNase-free water by pipetting. All RNA samples were stored at -80˚C until they were used.

The RNeasy mini kit (QIAGEN, Valencia, CA, USA) was also used for the isolation of RNA from cells. After collection of the cells in RNA later, the cells were disrupted by adding 600μL of Buffer RLT and pipetting to mix. The mixture was then homogenized with a Tissue Tearor (ThermoFisher Scientific, Wilmington, DE, US). Then 600μL of 70% ethanol was added and the solution was mixed by pipetting before adding 700μL of the solution to the RNeasy spin column. The spin column was centrifuged for 20 seconds at 10,000 rpm, the flow-through was discarded, and the remainder of the cell-containing solution was added to the column before centrifuging for 20 seconds at 10,000 rpm. The flow-through was discarded and 700μL of Buffer RW1 was added to the spin column and centrifuged for 20 seconds at 10,000 rpm. After discarding the flow-through, 500μL Buffer RPE was added to the spin column and the column was centrifuged for 20 seconds at 10,000 rpm followed by a second Buffer RPE wash and centrifugation for 1 minute at 10,000 rpm. The column was then placed in a new collection tube and dried by centrifuging for 1 minute at 10,000 rpm. The RNA was then eluted by placing the spin column in a 1.5mL tube, adding 30μL of RNAse-free water, and centrifuging for 1 minute at 10,000 rpm.

**Determination of RNA Integrity**. Determination of RNA Integrity was performed by using the Agilent 600 nano kit (Agilent Technologies, Santa Clara, CA). 1μL of the RNA 6000 Nano dye concentrate was added to 65μL of filtered gel matrix and was vortexed to mix. The gel-dye mix was then centrifuged for 10 minutes at 13,000 x g. A new RNA Nano chip was then placed on the chip priming station and 9μL of the gel-dye was added to the well marked by a white “G” in a black circle. The syringe of the chip priming station was set to 1mL and the chip priming station was then closed. The plunger of the syringe was then pressed down until it is held by the clip and was left for exactly 30 seconds before the clip was released. After waiting 5 seconds from the release of the clip, the plunger was slowly pulled back to the 1mL position. The chip priming station was then opened and the chip was removed. 9μL of the gel-dye mix was then added to the wells marked with a black “G”. Then 5μL of the RNA 6000 Nano marker was added to the well marked with a ladder symbol as well each of the 12 sample wells. 1μL of the RNA ladder was added into the well marked with a ladder symbol, and 1μL of each sample was added to the individual sample wells. The chip was then vortexed in the adapter of the IKA vortex mixer for 60 seconds at 2400 rpm. The chip was then analyzed with the Agilent 2100 bioanalyzer (Agilent Technologies, Santa Clara, CA).

**cDNA Synthesis.** Quantitative reverse transcription PCR was performed by generating cDNA from the collected RNA using the Omniscript RT kit (QIAGEN, Valencia, CA, USA) Complementary DNA was created by adding creating a master mix containing 2μL 10x Buffer RT, 2μL dNTP Mix at 5mM for each dNTP, 2μL Oligo-dT primer at 10μM, and 1μL of Omniscript Reverse Transcriptase per sample. 7μL of this mix was added to each tube in an 8-tube strip. 50ng of RNA was then added to each well, and the total volume in each tube was brought to 20μL with RNase-free water. The samples were then incubated in a thermal cycler for 60 minutes at 37˚C before holding at 4˚C.

**RT^2^ Profiler PCR Array.** RT^2^ Profiler^TM^ PCR Array Human Focal Adhesion (QIAGEN, Valencia, CA, USA) was used by by first collecting RNA and using the RT^2^ First Strand Kit to create cDNA. This was done by adding 25ng of RNA to 2μL Buffer GE and bringing the volume to 10μL with RNase-free water. This genomic DNA elimination mix was incubated at 42˚C for 5 minutes, and then immediately placed on ice for 1 minute. The reverse transcriptase mix was made by adding 4μL 5x Buffer BC3, 1μL Control P2, 2μL RE3 Reverse Transcriptase Mix, and 3μL RNase-free water for each sample. 10μL reverse-transcription mix was then added to each tube containing the DNA elimination mix, and the samples were mixed by pipetting. The samples were then incubated at 42˚C for 15 minutes followed by incubation at 95˚C for 5 minutes to stop the reaction. Each sample then had 91μL of RNase-free water added, and were stored at -20˚ until they were used.

To perform the PCR, first a master mix containing 1350μL 2x RT^2^ SYBR Green Mastermix, 102μL cDNA synthesis reaction and 1248μL RNase-free water was created. 25μL of this mix was then added to each well of the RT^2^ Profiler PCR array, and the plate was tightly sealed with optical thin-wall 8-cap strips. The plate was then centrifuged for 1 minutes at 1000 x g to remove bubbles, and inspected to ensure no bubbles were present. The PCR was run with a HotStart DNA *Taq* Polymerase activation for 10 minutes at 95˚C and 40 cycles with 15 seconds at 95˚C followed by 1 minute at 60˚C. The C_T_ values were determined and the RT^2^ qPCR Array Data Analysis spreadsheet was used to analyze the data.

**
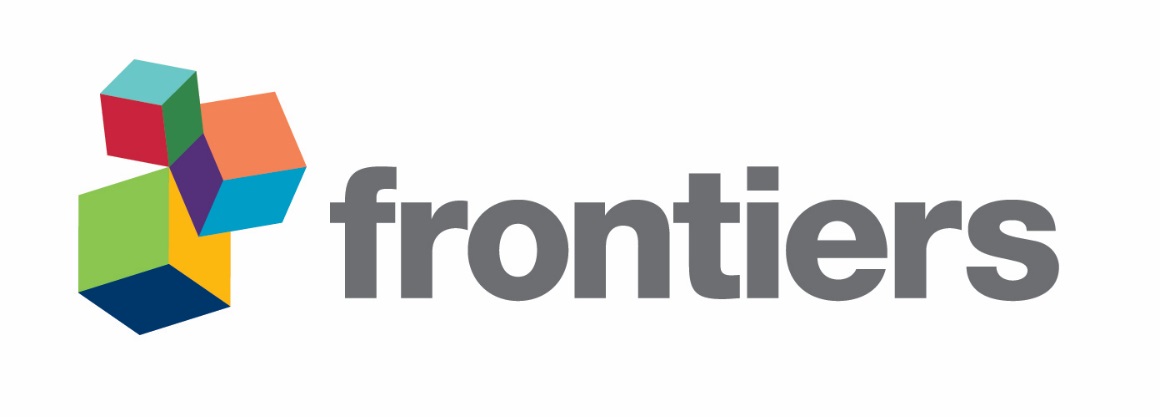
**
