# Supplementary figures and images for: Effects of Shear Stress on Production of FVIII and vWF in a Cell-Based Therapeutic for Hemophilia A
Source: Front Bioeng Biotechnol. 2021 Mar 1;9:639070. doi: 10.3389/fbioe.2021.639070 (PMC7957060; doi:10.3389/fbioe.2021.639070)

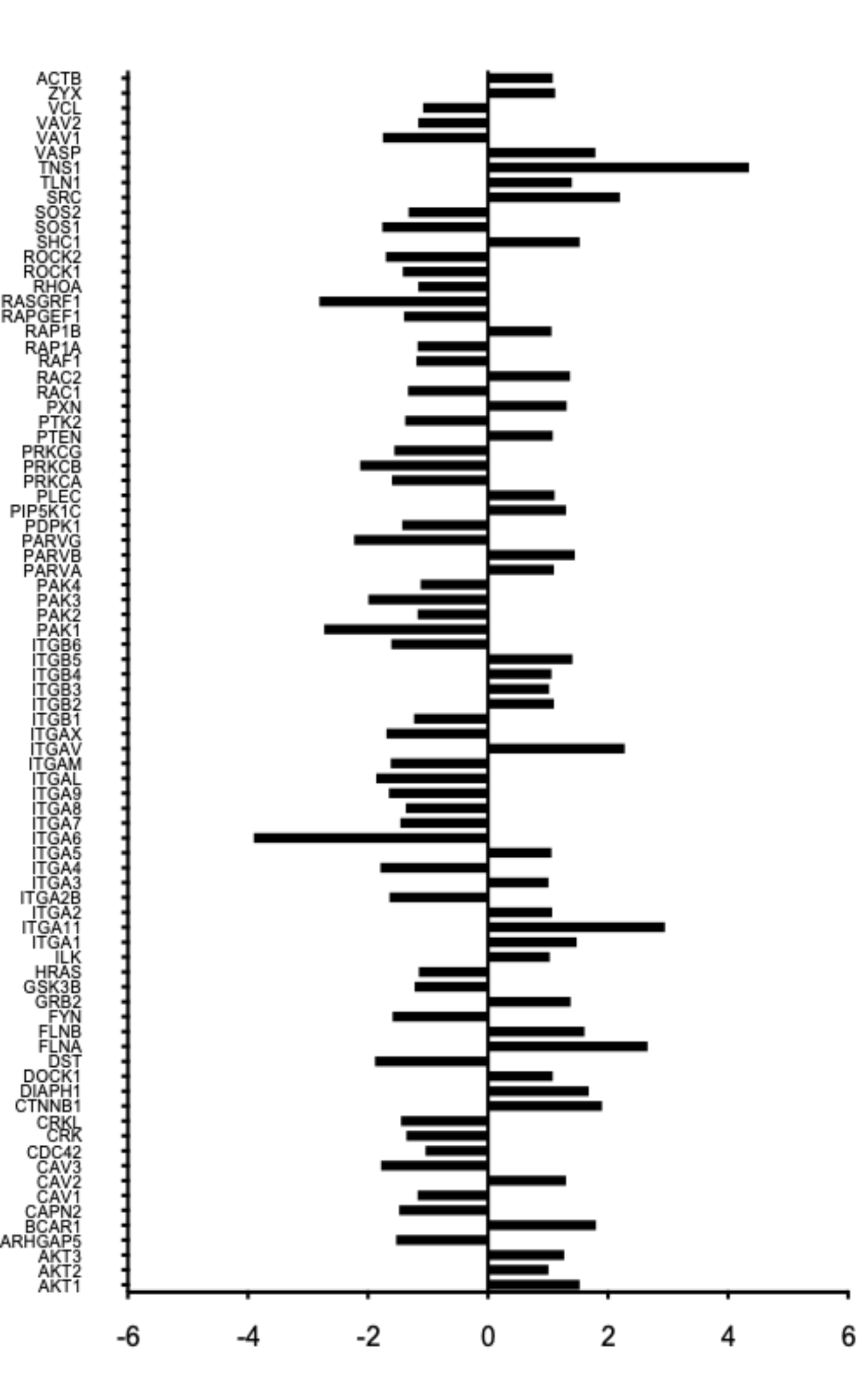

Supplement: Supplementary file 2 [file Image_1.TIF]
